# Outcomes of primary endoresection for choroidal melanoma

**DOI:** 10.1186/s40942-017-0096-5

**Published:** 2017-11-06

**Authors:** Andre A. C. Vidoris, Andre Maia, Marcia Lowen, Melina Morales, Jordan Isenberg, Bruno F. Fernandes, Rubens N. Belfort

**Affiliations:** 1 Department of Ophthalmology and Visual Sciences, Federal University of São Paulo, São Paulo, Brazil; 2 Department of Pathology, Federal University of São Paulo, São Paulo, Brazil; 3 Department of Ophthalmology, University of Montreal, Hôpital Maisoneuve-Rosemont, 5415 Boulevard de l’Assomption, Montreal, QC H1T 2M4 Canada; 4 Vegter Institute, Montreal, Canada

**Keywords:** Uveal melanoma, Endoresection, Plaque brachytherapy, Ocular tumor

## Abstract

**Purpose:**

To review long time treatment results in patients with small or medium sized choroidal melanoma who underwent surgical tumor endoresection as a primary treatment when plaque radiotherapy was unable and patients declined enucleation.

**Materials and methods:**

Patients were evaluated for best corrected visual acuity (BCVA), and underwent biomicroscopy, indirect ophthalmoscopy, retinography and ultrasound as well as the usual systemic workup. Study inclusion required the absence of scleral invasion or metastasis and an anterior margin not exceeding the pars plana or the ciliary body. Surgery consisted of a clear lens phacoemulsification with a PC-IOL, and a 23-gauge pars plana vitrectomy with anterior vitreous shave, lesional choroidal endodiathermy, followed by 23-gauge probe tumor endoresection and continuous endolaser. Patients were followed at post-operative 1 day, 1 week, 1, 3, and 6 months and then every 6 months with a complete ophthalmological exam including ultrasound biomicroscopy and systemic follow-up at 3, 6 and every 6 months thereafter.

**Results:**

Fourteen patients with choroidal melanoma were included the study. Pre-operative BCVA ranged from 20/20 to hand motion (HM): 20/20 (n = 2); 20/60 (n = 1); and HM (n = 10). Pathological analysis confirmed the diagnosis of uveal melanoma in all cases. Mean follow-up was 54.5 months (45–66 months) with a final BCVA ranging from 20/60 to HM: 20/60 (n = 1); 20/60 to 20/200 (n = 10); and HM (n = 2). The eye retention rate in our study was 100%. No intraocular recurrence was observed. One patient died 12 months after surgery from metastatic disease.

**Conclusion:**

Endoresection appears to be an acceptable alternative to enucleation for the treatment of posteriorly-localized uveal melanoma, with excellent local control and eye salvage rates.

**Electronic supplementary material:**

The online version of this article (doi:10.1186/s40942-017-0096-5) contains supplementary material, which is available to authorized users.

## Background

Uveal melanoma (UM) is the most common primary intraocular malignancy in adults, with a reported incidence of 6–7 cases annually per million people [1–3]. Approximately 80% of UMs occur in the choroid, and treatment of these tumors can either be radical—enucleation, for example—or conservative aiming to preserve the eye and as much vision as possible [2].

Conservative treatment approaches for choroidal melanoma include radiotherapy, phototherapy, and local resection. Plaque radiotherapy has been shown to be as effective as enucleation in preventing mortality from medium-sized choroidal melanomas [4], and it is thus the most common first-line treatment for small- and medium-sized tumors (height < 10 mm, diameter < 15 mm) [2]. Radiotherapy is generally administered to patients using ruthenium (Ru-106) or iodine (I-125) plaque brachytherapy, although it may be delivered by charged particles or in the form of stereotactic radiotherapy [2, 5]. For large UM tumors or those that do not respond well to radiotherapy, enucleation is the most frequent intervention [3, 5, 6].

Surgical endoresection or the local resection of posteriorly-localized UM offers several theoretical advantages over enucleation and radiotherapy [5, 7]. In contrast to enucleation, local resection of posterior UM aims to preserve vision and maintain cosmesis [7] and unlike radiotherapy, it has fewer long-term complications, including cataract and radiation retinopathy. Despite these advantages, to provide access to tumors located posteriorly and near the optic disc and/or macula, endoresection of choroidal melanomas involves the use of a vitrector to remove the tumor during pars plana vitrectomy. As such, potential complications of local resection include, vitreous hemorrhage, retinal detachment, and cataract.

Despite the concerns that local resection could cause intraoperative dissemination of tumor cells that can lead to recurrences [8], reports that have addressed this possibility have shown that rates of metastasis and mortality after enucleation, radiotherapy, or local resection of the tumor do not differ significantly [4, 6, 7, 9–13]. Leading some to propose endoresection as an alternative to both enucleation and brachytherapy for posteriorly-localized UM [8, 14–17].

The purpose of this study is to evaluate endoresection as a primary treatment for choroidal melanoma.

## Materials and methods

Between January 2012 and December 2013, fourteen patients with choroidal melanoma—diagnosed by indirect ophthalmoscopy and ultrasound —underwent complete ophthalmologic evaluation and systemic workup for metastasis. Endoresection was presented as an alternative to enucleation; plaque brachytherapy—which is not always covered by the public healthcare system in Brazil—was not an available treatment option for the patients in this study.

All patients underwent endoresection surgery that was performed by the same surgeon (A.M.). The inclusion criteria were as follows: location of the posterior margin of the tumor > 3 mm from the optic disc; absence of scleral invasion of the tumor; absence of metastasis; and anterior margin of the tumor not exceeding the pars plana and ciliary body. Patients were fully informed about the disease as well as the experimental treatment and the potential risks thereof. Enucleation as an alternative treatment was discussed with all patients. All relevant aspects of the procedure were explained to the patients, and informed written consent was obtained from all patients.

Pre-operative exams for all patients included: best-corrected visual acuity (BCVA; Snellen); full ophthalmic examination; A- and B-scan ultrasonography, with measurements of the maximal tumor thickness and basal diameter (AP: anterior–posterior; LL: lateral–lateral); fundus photography; and screening for metastatic disease, which included serum biochemistry analysis with liver function tests, abdominal echography, abdominal computed tomography (CT), and chest radiography.

The surgery started with a standard clear lens phacoemulsification and posterior chamber intraocular lens (PCIOL) implantation in the bag. A 23-gauge pars plana vitrectomy with valved trocars (Constellation, Alcon, Fort Worth, TX, USA) was performed. Diluted triamcinolone acetonide was injected into the mid-vitreous cavity to stain the cortical vitreous and assist with its complete removal, which involved peeling the posterior hyaloid by applying a gentle vacuum with the probe. A complete anterior vitreous shave was performed under scleral depression and chandelier illumination (Synergetics Inc, St. Charles, MO, USA). Retinectomy of 180^o^ at the ora serrata adjacent to the tumor was performed with the cutter.

Choroidal endodiathermy was performed around the lesion, followed by endoresection of the tumor with the 23-guage probe. Intraocular pressure was kept at 60 mmHg during this step to prevent bleeding. Continuous endolaser (500 mW) was performed on the scleral bed. The retina was reattached with perfluorocarbon liquid. At the end of the case, a fluid-air exchange was made and the eye was filled with silicone oil (5000 cst-DORC, The Netherlands). Next, closure of the sclerotomies and conjunctiva was performed using 7-0 Vicryl sutures (polyglactin 910; Ethicon Inc, Johnson and Johnson, Somerville, NJ, USA).

All patients were evaluated for BCVA, and all underwent slit lamp microscopy, indirect ophthalmoscopy, and retinography at 1 day, 1 week, 1, 3, and 6 months post-surgery, and then every 6 months thereafter. UBM (ultrasound biomicroscopy) was performed every 6 months for 2 years to evaluate the sclera for tumor insertion around the vitrectomy ports. The systemic workup with blood exams and liver imaging was performed at the time of diagnosis, as well as every 3 months for 1 year and every 6 months in the subsequent post-operative years.

All material collected during vitrectomy for each case was centrifuged, prepared as a cell block, formalin-fixed, paraffin-embedded, and submitted for pathology studies. Our technique as compared to other studies can be seen in Additional file 1: videos S1 with videos oneline in Additional file 2: Table S2.

## Results

**Fig. 1:**
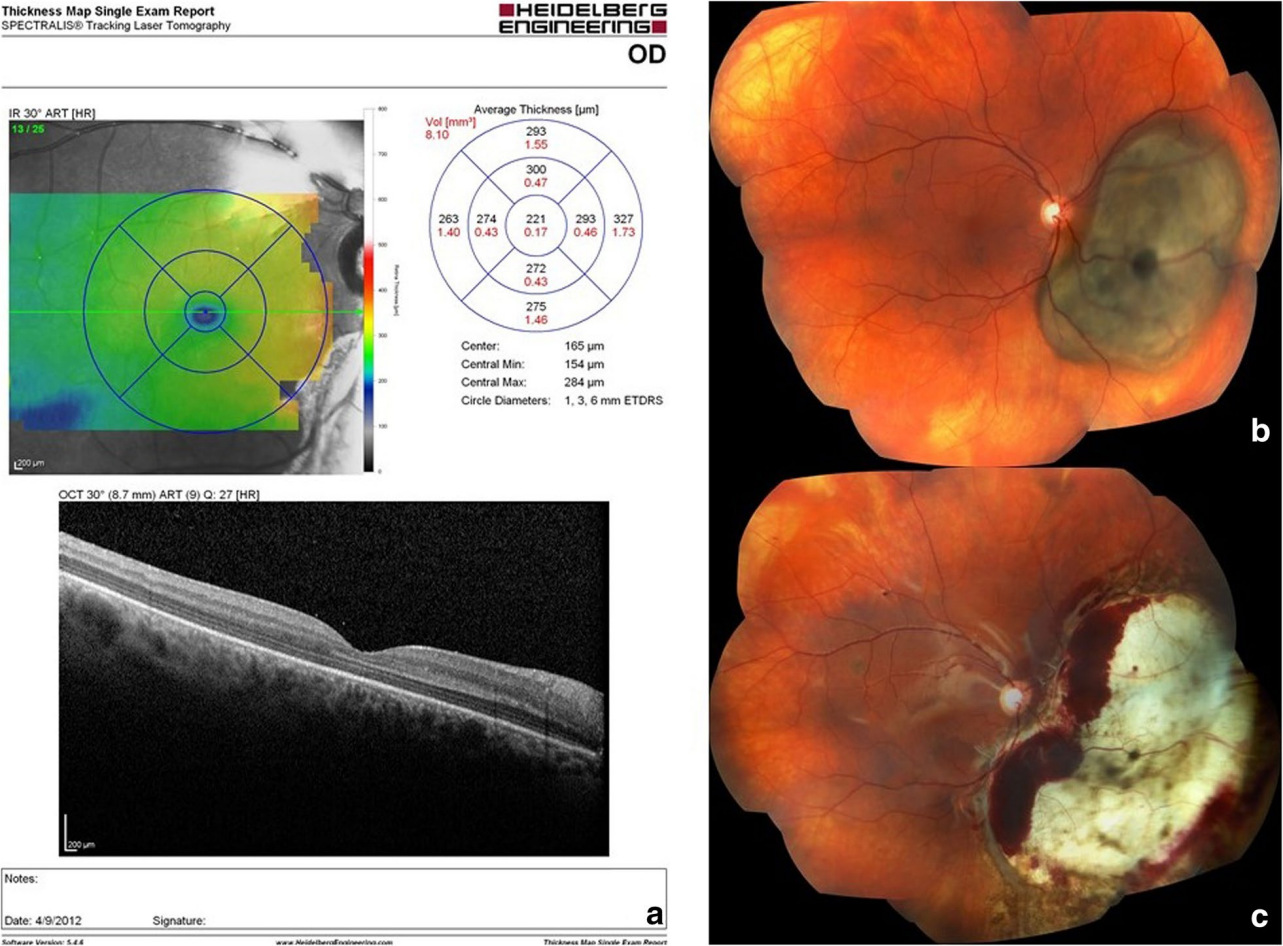
Same patient 28-day post-operative macular OCT (**a**) and pre-operative (**b**) and 28 days post-operative (**c**) color fundus photograph

**Fig. 2:**
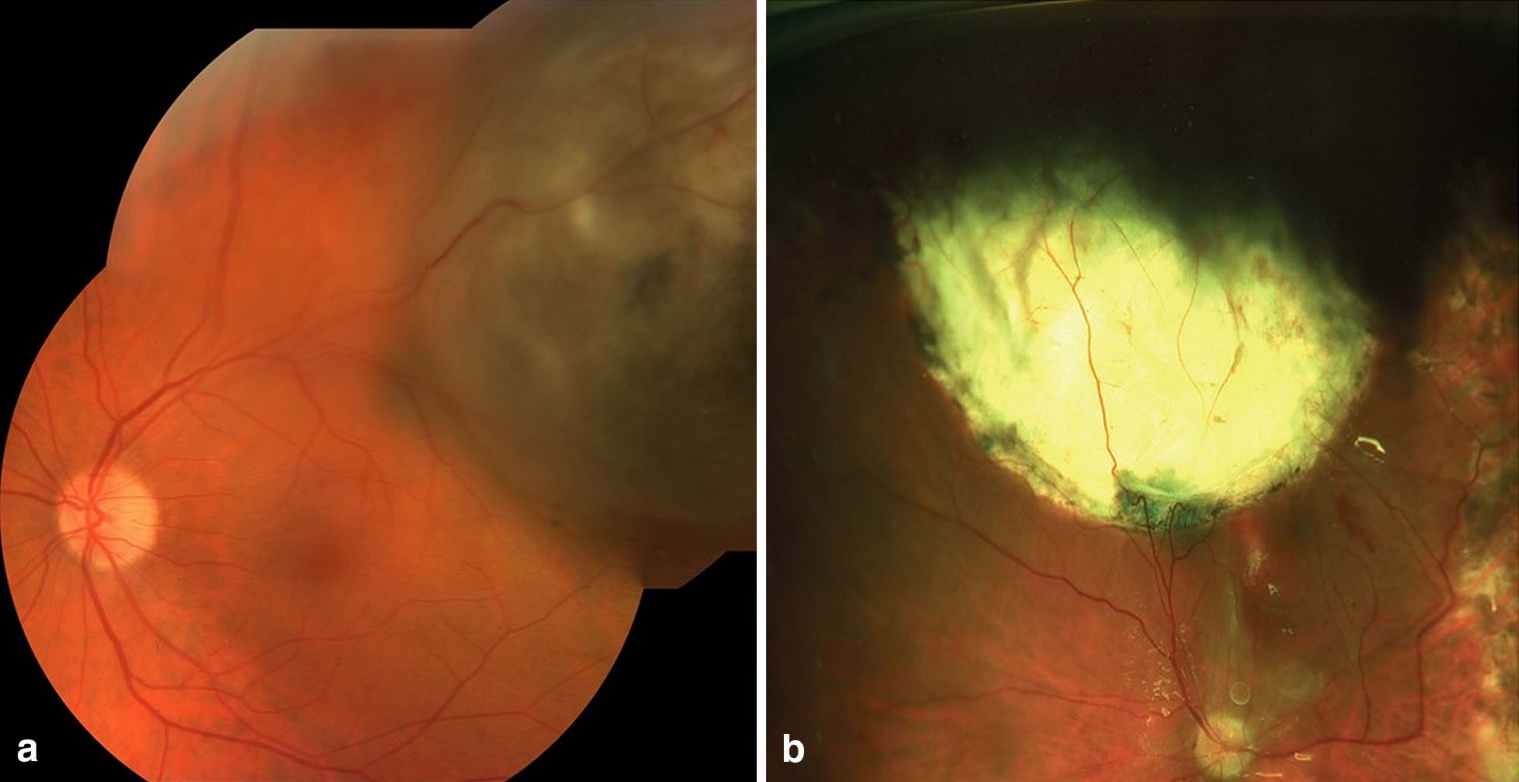
Color fundus photograph of same patient pre-operative (**a**) and 28 days post-operative (**b**)

Fourteen patients, including eight females and five males, were post-operatively followed for an average of 48 ± 6.4 months (range: 12–60 months). Mean age at the time of surgery was 50.3 ± 8.6 years (range: 40–66 years). Average tumor thickness was 6.05 ± 1.94 mm (range: 3.3–9.7 mm). The tumor basal AP diameter averaged 11.2 ± 4.16 mm (range: 5.2–18.8 mm); basal L–L diameter averaged 15.98 ± 4.76 mm (range: 4.3–23 mm). B-scan revealed that eight cases had dome-shaped tumors, while five cases had mushroom-shaped tumors. A-scan showed angle kappa for all cases. The tumor location was in the left eye in six cases and in the right eye in seven cases (Fig. 1); twelve were posterior to the equator (Fig. 2), while one reached the ciliary body anterior to the equator.

**Fig. 3:**
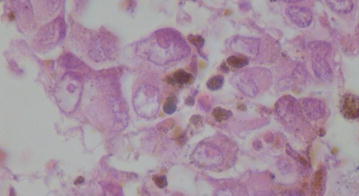
mixed cell type uveal melanoma though predominantly ellipsoid with characteristic atypia and pleiomorphisms

Pre-operative BCVA ranged from 20/20 to hand motion (HM). Distribution was as follows: 20/20 (n = 2); 20/60 (n = 1); HM (n = 10). Pathological analysis confirmed the diagnosis of UM in all cases. Tumors were classified as mixed cell type (n = 12) (Fig. 3) or spindle cell type (n = 1).

With respect to surgical complications, one patient had post-operative macular bleeding that resolved in 1 month (in this case the tumor had a distance of 3 mm from the macula and the surgeon did not use endodiathermy near the macular area). One patient had elevated intraocular pressure (IOP) and required non-invasive glaucoma surgery (cyclophotocoagulation). Two patients presented with retinal detachment during follow-up, one had air in the first surgery and received silicon oil on the second procedure.

In a mean follow-up of 54.5 months, final BCVA ranged from 20/60 to HM, as follows: 20/60 (n = 1), vision between 20/60 and 20/200 (n = 10), and HM (n = 2; both with macular tumors).

The eye retention rate in our study was 100%. One patient presented with liver nodules that were biopsied and confirmed to be metastatic disease, and this patient died 12 months after diagnosis. Another patient died of unrelated disease (myocardial infarction) during the follow-up period and was excluded from follow-up.

## Discussion

UM treatment goal is to control the melanoma and avoid enucleation, even is final vision is poor. Plaque brachytherapy is the gold standard for treating small- and medium-sized tumors, while enucleation is still the default procedure for large choroidal melanomas.

The primary criticism of using endoresection as a first-line treatment alternative to brachytherapy is that it may cause intraoperative dissemination of tumor cells, leading to local recurrences or an increased risk of systemic disease. Despite this fear, there is a lack of evidence to support a higher frequency of local tumor recurrence in cases treated by endoresection using recent vitrectomy technique versus brachytherapy. Even though Damato showed 19% recurrence in 12 cases of tumor recurrence out of 61 patients treated with endoresection they did not employ valvulated trocars nor did they control of intraocular pressure per-op [2]. A 2013 study by Caminal et al. compared the long-term surgical outcomes in patients with choroidal melanoma who were treated using either endoresection or I-125 brachytherarpy; the study’s authors concluded that endoresection for choroidal melanoma was an effective treatment modality in selected cases of posterior choroidal melanomas, with outcomes similar to those obtained with iodine-125 brachytherapy [18]. Karkhaneh et al. came to similar conclusions in their 2007 report of 20 patients with posterior choroidal melanoma who were treated via endoresection [5]. An earlier study by Char et al. reported that local resection led to higher ocular retention rates and better visual outcomes than radiation in UM cases with thick, posteriorly-localized tumors [19]. To date to the best of our knowledge there are no randomized clinical trial comparing brachytherapy to endoresection.

Since 2011 ruthenium plaque brachytherapy defunded in Brazil’s public healthcare system [18]. In this study, we wanted to consider primary endoresection as a first-line treatment alternative to plaque brachytherapy to avoid enucleation.

There were no case of local recurrence during the follow-up period (54.5 months), the treated eye was saved in all cases (no enucleations) and there was no severe bleeding during surgery, despite previous reports that have highlighted this possible complication [5]. Final visual acuity in our patients was poor, but the size and location of the tumors must be taken into consideration; patients with large tumors and tumors in the posterior pole, especially those located on the macula, are expected to have a poor visual prognosis. When compared to a large cohort of plaque radiotherapy patient most with small or medium sized tumors had visual acuity better than 20/200 at 36 months, slightly better than ours. [20].

The patient with the largest tumor in this series 23 mm at the base at 15 mm thick was lost to follow up in the immediate post-operative period and presented with liver metastasis 6 months post-op. Prior to surgery there was no metastatic disease and surgical margins were negative. It is unclear whether this is a case of iatrogenic-intravitrous dissemination or were circulating malignant cells (CMC) present with a seeded liver pre-op. Given the constraints of the Brazilian public health care system CMC detection and chromosomal analysis were not possible. There are several reports in the literature describing intravitreal invasion by melanoma cells from a collar button choroidal melanoma which began shedding pigmented debris into the vitreous, though this was not found noted on pathological analysis of any samples in this series [21]. We have not observed any intrascleral invasion, maybe due to lasering of the scleral bed after removing the tumor [22].

Damato has observed 22% of retinal detachment in his series, we had 2 cases (15%) of retinal detachment. Those detachments happened on the first 5 cases treated and we believe it could be related to surgeon learning curve. After those cases we started performing lens extraction to allow a better vitreous removal [23]. Overall our outcomes are comparable to the literature (Table 1).

**Table 1:** Study outcome as compared to other endoresection study

Authors	Year	Eyes	Mean follow up time (months)	Liver metastasis (% of cases)
Damato	1998	52	20	2
Caminal	2013	27	70.52	4
Karkhaneh	2007	20	47.1	5
Vidoris	2017	14	54.5	7

## Conclusion

In this 54.5-month long-term follow-up, endoresection appears to be an acceptable alternative to enucleation for the treatment of posteriorly-localized UM, with excellent local control and eye salvage rates. Short-term complications were related to the surgical procedure and not to the tumor itself (i.e. tumor recurrence or metastasis). In addition, pathological evaluation of the tumors allowed the diagnostic to be confirmed in all cases. Very long-term follow-up (more than 15 years) will be necessary to assess the technique’s impact on patient mortality rates but on 54.5 months follow up there seems to be no increase in mortality.

## Additional files

**Additional file 1.** Video of three of the 14 surgical cases presented.

**Additional file 2.** Comparison of various endoresction techniques in the literature.

## References

[1] Egan KM (1988). Epidemiologic aspects of uveal melanoma. Surv Ophthalmol.

[2] Hadden PW, Hiscott PS, Damato BE (2004). Histopathology of eyes enucleated after endoresection of choroidal melanoma. Ophthalmology.

[3] Fernandes BF (2007). Detection of circulating malignant cells in patients with uveal melanoma. Arq Bras Oftalmol.

[4] Diener-West M (2001). The COMS randomized trial of iodine 125 brachytherapy for choroidal melanoma, III: initial mortality findings. COMS Report No. 18. Arch Ophthalmol.

[5] Karkhaneh R (2007). Long-term surgical outcome of posterior choroidal melanoma treated by endoresection. Retina.

[6] Kertes PJ, Johnson JC, Peyman GA (1998). Internal resection of posterior uveal melanomas. Br J Ophthalmol.

[7] Shields CL, Shields JA (2004). Recent developments in the management of choroidal melanoma. Curr Opin Ophthalmol.

[8] Gündüz K, Bechrakis NE (2010). Exoresection and endoresection for uveal melanoma. Middle East Afr J Ophthalmol.

[9] García-Arumí J (2001). Vitreoretinal surgery and endoresection in high posterior choroidal melanomas. Retina.

[10] Foulds WS, Damato BE, Burton RL (1987). Local resection versus enucleation in the management of choroidal melanoma. Eye (Lond).

[11] Zimmerman LE, McLean IW (1979). An evaluation of enucleation in the management of uveal melanomas. Am J Ophthalmol.

[12] Zimmerman LE, McLean IW, Foster WD (1978). Does enucleation of the eye containing a malignant melanoma prevent or accelerate the dissemination of tumour cells. Br J Ophthalmol.

[13] The Collaborative Ocular Melanoma Study (1998). (COMS) randomized trial of pre-enucleation radiation of large choroidal melanoma II: initial mortality findings. COMS report no. 10. Am J Ophthalmol.

[14] Peyman GA, Cheema RA, Lagouros PA (2001). Endoresection of a ciliochoroidal melanoma. Can J Ophthalmol.

[15] Damato B (1998). Endoresection of choroidal melanoma. Br J Ophthalmol.

[16] Peyman GA, Charles H (1988). Internal eye wall resection in the management of uveal melanoma. Can J Ophthalmol.

[17] Peyman GA, Cohen SB (1986). Ab interno resection of uveal melanoma. Int Ophthalmol.

[18] Caminal JM (2013). Endoresection versus iodine-125 plaque brachytherapy for the treatment of choroidal melanoma. Am J Ophthalmol.

[19] Char DH, Miller T, Crawford JB (2001). Uveal tumour resection. Br J Ophthalmol.

[20] Shields CL, Shields JA, Cater J, Gündüz K, Miyamoto C, Micaily B, Brady LW (2000). Plaque radiotherapy for uveal melanoma: long-term visual outcome in 1106 consecutive patients. Arch Ophthalmol.

[21] Robertson DM, CampbelL RJ (1997). Intravitreal invasion of malignant cells from choroidal melanoma after brachytherapy. Arch Ophthalmol.

[22] Damato B (2001). Intrascleral recurrence of uveal melanoma after transretinal “endoresection”. Br J Ophthalmol.

[23] Konstantinidis L (2014). Long-term outcome of primary endoresection of choroidal melanoma. Br J Ophthalmol.

