# Supplementary material for: Outcomes of primary endoresection for choroidal melanoma
Source: Int J Retina Vitreous. 2017 Nov 6;3:42. doi: 10.1186/s40942-017-0096-5 (PMC5674241; doi:10.1186/s40942-017-0096-5)
Supplement: Supplementary file 2 — Additional file 2. Comparison of various endoresction techniques in the literature. [file 40942_2017_96_MOESM2_ESM.docx]

| Author | Year | Technique |
| --- | --- | --- |
| Damato | 1998 | The current technique involves vitrectomy, retinal incision over or peripheral to the tumour, haemostasis by raising intraocular pressure and by moderate hypotensive anaesthesia, choroidal incision around tumour, endoresection with vitrector, endodiathermy to bleeding points and residual tumour, fluid-air exchange to reattach retina, endolaser to achieve retinal adhesion around the coloboma and destroy residual tumour in the sclera, silicone oil injection with removal after 12 weeks, cryotherapy to the sclerotomies, and adjunctive ruthenium plaque radiotherapy in selected cases |
| Caminal | 2013 | 20- to 23-gauge vitrectomy using a panoramic viewing system, followed by posterior hyaloid dissection, large retinotomy anterior to the tumor, and laser endophotocoagulation with 600-800 mW directed 2 mm beyond the tumor margins using the continuous mode of a 532-nm laser. The melanoma lesion was removed with the vitrectomy probe using a bimanual technique to separate and protect the retina. Intraoperative choroidal bleeding was controlled by temporarily raising the intraocular pressure (IOP) to 60-70 mm. In all cases, patient blood pressure remained within normal limits during the procedure. Tumor excision was performed from the tumor apex to the scleral bed. Cellular remnants at the scleral bed were photocoagulated with high doses (800-1000 mW) of continuous-mode laser endophotocoagulation at 532 nm. The retina was reattached with perfluorocarbon liquid. Laser retinopexy endophotocoagulation was performed at the margins of the retinotomy, and this procedure was followed by fluid-air exchange and silicone oil-air exchange (1300-5000 centistokes). |
| Karkhaneh | 2007 | The surgical technique included three-port standard vitrectomy, followed by posterior hyaloid dissection, retinal coagulation (cauterization) 2 mm beyond the tumor margins using endodiathermy, and retinotomy at the apex of the tumor. The retina was held away from the vitrectomy probe, and tumor excision was begun at the apex of the tumor until the scleral bed inside the circle delineated by diathermy was free of tumor. Intraoperative bleeding was controlled by temporarily raising the infusion bottle to elevate the intraocular pressure. If the tumor was under the fovea, peripheral retinotomy was performed, and the tumor was removed under a retinal flap; the cellular remnants at the scleral bed were photocoagulated with the endolaser probe. The retina was reattached with perfluorocarbon liquid and air. Laser retinopexy endophotocoagulation was performed at the limit of the retinotomy, followed by fluid–air exchange and silicone oil exchange. Silicone oil was used as internal tamponade because it limits bleeding to the surgical coloboma, allowing the surgeon good postoperative control. For one patient (Case 15), SF_6_ gas was used as internal tamponade because there was slight bleeding during choroidectomy and no bleeding was observed at the margin of resection after completion of laser coagulation and fluid–air exchange |
| Vidoris | 2017 | see above |
